# Cardiac Aftermath of Gestational Diabetes—From Intrauterine Impact to Lifelong Complications: A Systematic Review

**DOI:** 10.3390/jdb13040044

**Published:** 2025-12-08

**Authors:** Sophia Tsokkou, Ioannis Konstantinidis, Antonios Keramas, Vasileios Anastasiou, Alkis Matsas, Maria Florou, Alexandra Arvanitaki, Emmanouela Peteinidou, Theodoros Karamitsos, George Giannakoulas, Themistoklis Dagklis, Theodora Papamitsou, Antonios Ziakas, Vasileios Kamperidis

**Affiliations:** 1 1st Department of Cardiology, School of Medicine, Faculty of Health Sciences, Aristotle University of Thessaloniki, 54124 Thessaloniki, Greece; stsokkou@auth.gr (S.T.); antonios@auth.gr (A.K.); vasianas44@gmail.com (V.A.); emma2405@windowslive.com (E.P.); tkaramitsos@auth.gr (T.K.); ggiannakoulas@auth.gr (G.G.); aziakas@auth.gr (A.Z.); 2 Laboratory of Histology-Embryology, Department of Medicine, Faculty of Health Sciences, Aristotle University of Thessaloniki, 54124 Thessaloniki, Greece; ikonsc@auth.gr (I.K.); thpapami@auth.gr (T.P.); 3 Laboratory of Experimental Surgery and Surgical Research ‘N.S. Christeas’, Medical School, National and Kapodistrian University of Athens, 11527 Athens, Greece; amatsas@med.uoa.gr; 4 Second Department of Pediatric Surgery, Aristotle University of Thessaloniki Papageorgiou General Hospital, 56403 Thessaloniki, Greece; flwrou.mar@gmail.com; 5 Second Department of Cardiology, School of Medicine, Faculty of Health Sciences, Aristotle University of Thessaloniki, 54124 Thessaloniki, Greece; alexandra.arvanit@gmail.com; 6 Third Department of Obstetrics and Gynecology, School of Medicine, Faculty of Health Sciences, Aristotle University of Thessaloniki, 54642 Thessaloniki, Greece; dagklis@auth.gr

**Keywords:** gestational diabetes, fetal cardiac function, fetal cardiomyopathy, echocardiography, cardiovascular risk, pregnancy

## Abstract

**Background**. Gestational diabetes mellitus (GDM) induces maternal hyperglycemia, which may alter fetal cardiac structure and function, increasing short- and long-term cardiovascular risks. **Purpose**. To systematically review the evidence on the fetal cardiac structural and functional effects of GDM, to explore the diagnostic role of novel imaging and biochemical biomarkers, and to summarize the long-term cardiovascular complications associated with GDM. **Materials and Methods**. A systematic search of PubMed, Scopus, and Cochrane Library was conducted according to the PRISMA guidelines. All studies comparing cardiac outcomes in GDM and non-GDM pregnancies were included. Data on myocardial hypertrophy, diastolic and systolic function, imaging modalities, and biomarkers were extracted and qualitatively synthesized. **Results**. A total of twelve eligible studies were identified. Fetal cardiac hypertrophy and diastolic and early systolic dysfunction are common among GDM pregnancies and can be detected by dual-gate Doppler and speckle-tracking echocardiography. Abnormalities are observed in indices such as the myocardial performance index, E/A, E/e′ ratios, and global longitudinal and circumferential strain in fetuses and may persist in the neonatal period. Alterations may be more pronounced for the right ventricle compared to the left. Septal hypertrophy is associated with elevated umbilical cord pro-brain natriuretic peptide. The risk of early-onset cardiovascular disease in the progeny of diabetic mothers is 29% higher, as evidenced by population-based cohort data. **Conclusions**. GDM is linked to fetal cardiac remodeling and an increased long-term cardiovascular risk. Early detection and customized interventions to reduce adverse outcomes may be achieved by integrating advanced echocardiographic techniques and biomarkers into prenatal surveillance.

## 1. Introduction

Gestational diabetes mellitus (GDM) is a disease with growing prevalence that has garnered a lot of attention over the last years, with its diagnostic criteria continuously evolving. Initially, GDM was defined by the National Diabetes Data Group as glucose intolerance first recognized during pregnancy [1]. However, modern diagnostic criteria, set by the International Association of Diabetes and Pregnancy Study Groups, focus on maternal glycemia’s direct impact on adverse perinatal outcomes [2].

Maternal hyperglycemia has been increasingly linked to structural and functional alterations in fetal cardiac development, such as hypertrophic remodeling and impaired diastolic function. These abnormalities are considered to arise due to disrupted metabolic signaling and oxygen supply during critical stages of heart formation [3]. Evidence indicates a continuous, linear relationship between maternal glucose levels, increased birth weight, and increased cord-blood serum C-peptide levels, reinforcing the importance of screening and intervention [4]. Thus, novel imaging tools are emerging for the early detection of fetal cardiac abnormalities.

In addition, GDM exposure may adversely impact long-term offspring outcomes. Several studies show that prenatal exposure to maternal hyperglycemia contributes to increased adiposity, insulin resistance, glucose intolerance, and a higher predisposition to type 2 diabetes in adulthood [5]. These findings further support the hypothesis that intrauterine hyperglycemia can negatively affect fetal pancreatic function, increasing lifelong cardiometabolic risks [6]. Preventive strategies aimed at mitigating the intergenerational effects of GDM are crucial, while optimizing diagnostic and treatment approaches remains essential to improving both maternal and neonatal health outcomes.

The current study aims to scrutinize the structural and functional cardiac effects of GDM on the fetus, explore its role in long-term cardiovascular disease, and investigate the value of novel imaging biomarkers for early and accurate diagnosis of cardiac structural alterations.

## 2. Materials and Methods

To ensure transparency and methodological rigor, the current systematic review was implemented in accordance with the Preferred Reporting Items for Systematic Reviews and Meta-Analyses (PRISMA) guidelines. For the identification of appropriate studies published up to 15 June 2025, a thorough search was conducted across numerous electronic databases, such as PubMed (MEDLINE), Scopus, and Cochrane Library. The strategic search was developed using controlled vocabulary and keyword variations in accordance with the Boolean query: (Gestational diabetes mellitus OR GDM) AND (fetal cardiac anomalies OR fetal cardiomyopathy). The reference lists of included studies were also screened and relevant review articles for additional eligible studies were included to maximize sensitivity. To more explicitly define the research question, a population, intervention, comparator, and outcome (PICO) table was developed (Table 1). This systematic review has been registered in the PROSPERO database (ID: CRD420251064919).

### 2.1. Eligibility Criteria

Studies were included if they met the following criteria:

- Peer-reviewed observational (cohort, case–control, cross-sectional) or interventional (randomized controlled trials) studies.
- Population including pregnant individuals diagnosed with GDM based on established clinical criteria (either diagnosed during or prior to pregnancy).
- Studies assessing fetal heart anomalies, including structural defects and functional impairments resulting in fetal cardiomyopathy.
- Studies providing quantitative data on the prevalence or risk of fetal cardiac anomalies in pregnancies affected by GDM.

Animal studies, editorials, and studies lacking relevant outcome data and papers not written in the English language were excluded.

### 2.2. Study Selection

**Figure 1:**
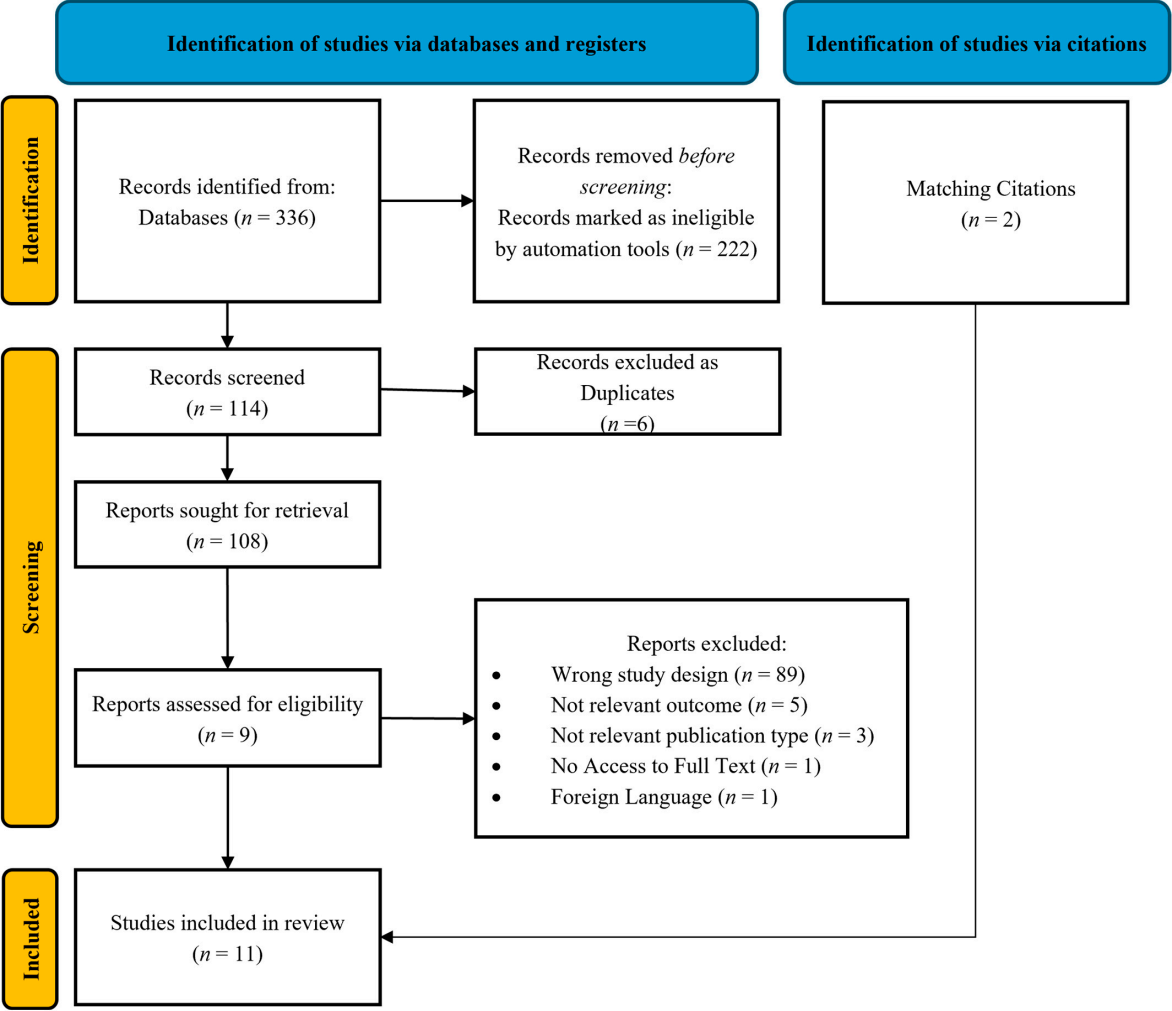
Prisma flow diagram.

All retrieved records underwent a two-step screening process by two independent reviewers (S.T. and I.K.). The initial screening involved a review of the title and abstract, followed by a full-text assessment of potentially eligible studies. Disagreements between reviewers were resolved through consensus or consultation with a third reviewer (A.M.). The final set of included studies was documented using a PRISMA flow diagram (Figure 1).

### 2.3. Data Extraction

Data extraction was conducted using a standardized form capturing study characteristics (author, year, design, sample size), population details (maternal age, gestational age, GDM diagnostic criteria), fetal cardiac outcomes, and key results.

### 2.4. Quality Assessment of Study Using GRADE and Newcastle–Ottawa Scale (NOS)

To ensure proper methodological evaluation rigor and ensure the reliability of the studies included in this analysis, two established frameworks were used—the Grading of Recommendations, Assessment, Development, and Evaluations (GRADE) system, as well as the Newcastle–Ottawa Scale (NOS). A detailed description of the quality assessment tools is available in the Supplementary Material.

## 3. Results

### 3.1. PRISMA Flow Diagram

A total of 336 studies were initially identified, of which 11 were finally included in the current systematic review (Figure 1).

### 3.2. Quality Assessment Using GRADE and NOS System Evaluation

#### 3.2.1. GRADE System Evaluation

Most of the included studies were considered of moderate quality. Observational cohort studies with long-term follow-up and rigorous methodology were upgraded to high-quality evidence, such as that of Yu et al., 2019 [7], which provided robust epidemiological insights into maternal diabetes and offspring cardiovascular risk. Studies that employed standardized echocardiographic techniques with well-defined control groups, such as those of Chen et al., 2022 [8] and Miranda et al., 2018 [9], also received high ratings. However, studies with small sample sizes or a lack of blinding were downgraded due to imprecision and potential bias, placing them in the low-quality category (Supplementary Table S1).

#### 3.2.2. New Ottawa Scale (NOS) Evaluation System

The NOS (Supplementary Table S2) was used to assess the methodological quality of the observational studies. Out of the included studies, it was revealed that studies such as that of Yu et al., 2019 [7] scored 10/10, reflecting robust cohort designs with longitudinal follow-up and epidemiological validity. Similarly, the studies of Chen et al., 2022 [8] and Miranda et al., 2018 [9] were highly rated due to their standardized echocardiographic techniques and structured neonatal assessments. On the contrary, moderate-scoring studies such as those of Bogo et al., 2021 [10], Kulkarni et al., 2017 [11], and Bhorat et al., 2014 [12] provided valuable insights but lacked either comprehensive follow-up or full methodological transparency.

### 3.3. Main Findings

The main findings of this analysis are comprehensively summarized in Table 2, presenting an overview of structural and functional cardiac alterations in fetuses and neonates of diabetic mothers, along with their potential long-term cardiovascular consequences. The table consolidates data from multiple studies, detailing fetal myocardial hypertrophy, diastolic dysfunction, and altered myocardial performance indices observed across different gestational stages. It highlights the predictive value of advanced imaging modalities, biochemical markers, and maternal metabolic control in determining neonatal outcomes.

### 3.4. Fetal Myocardial Remodeling in Diabetic Pregnancies: Structural and Functional Insights

Tejaswi G.M. et al. examined the fetal cardiac changes in diabetic and non-diabetic mothers from 24 weeks gestation until the neonatal period. The study concludes that fetal myocardial hypertrophy (MH), specifically thickening of the interventricular septum (IVS), right ventricle (RV), and left ventricle (LV), is significantly more pronounced in cases of poor maternal glycemic control. Myocardial remodeling progresses throughout gestation and persists into the neonatal period, particularly among fetuses of mothers with suboptimal glucose regulation [13]. Functionally, the study identified impaired systolic tissue annular velocity across the IVS and RV at term. Notably, neonatal cardiac function remained largely preserved despite the observed fetal MH, suggesting that in utero structural adaptations may not necessarily translate into postnatal cardiac dysfunction and long-term cardiac remodeling [13].

Adding to this research, Chen Y. investigated the effects of well-controlled GDM on fetal cardiac geometry and function using Fetal-Heart Quantification; an advanced speckle-tracking echocardiographic technique. In mothers with good glycemic control, fetal cardiac geometry remained mostly unaffected, with no significant abnormalities in ventricular wall thickness or cardiac sphericity indices. However, 60% of fetuses exposed to GDM exhibited at least one type of ventricular contractility dysfunction, with the RV being disproportionately affected in 50% of total cases. The most prevalent abnormality was transverse contractility dysfunction, observed in 35% of GDM fetuses, followed by global contractility impairment, observed in 25%, and longitudinal contractility abnormalities, observed in 21.3%. The LV was affected as well, with abnormal transverse contractility (18.8%) being more frequent than global or longitudinal dysfunction. The findings suggest that even when well-managed, maternal diabetes can impact fetal cardiac function, particularly the RV contractility, highlighting the clinical significance of conducting routine fetal cardiac assessments in mothers with GDM throughout the gestational period [8].

**Table 2:** Comprehensive summary of studies evaluating fetal cardiac function and long-term cardiovascular outcomes in maternal diabetes.

Category	Study Objective	Study Group	Key Measurements	Main Findings
Bhorat et al., 2014 [12]	Assess cardiac function in fetuses of poorly controlled gestational diabetics and its impact on perinatal outcomes.	29 pregnant women with severe gestational diabetes vs. 29 healthy controls.	Mod-MPI, E/A ratios via Doppler echocardiography.	There is significant impairment of cardiac function in fetuses of poorly controlled gestational diabetics. Mod-MPI and E/A ratio have the potential to improve fetal surveillance in diabetic pregnancies.
Mohsin M. et al., 2019 [14]	The purpose of this study was to assess fetal cardiac function in normal fetuses (control group) compared to those who are exposed to gestational diabetes mellitus using different echocardiographic measurements, and to explore the application of left atrial shortening fractioning determination of fetal diastolic function with gestational diabetes mellitus.	50 women with gestational diabetes and 50 women with a healthy pregnancy were included in the study.	Fetal echocardiography was performed and structural as well as functional fetal cardiac parameters were measured.	Fetuses of gestational diabetic mothers have altered cardiac function even in the absence of septal hypertrophy, and left atrial shortening fraction can be used as a reliable alternate parameter in the assessment of fetal diastolic function.
Bogo et al., 2016 [10]	To evaluate cardiac function and structural changes in children of diabetic mothers in the fetal and neonatal period using Doppler-echocardiographic data.	48 children of mothers with clinically compensated GDM, single pregnancies, and no malformations.	Myocardial thickness, shortening fraction, left ventricular (LVMPI) and right ventricular (RVMPI) myocardial performance index, and mitral and tricuspid valve E/A ratio were evaluated in 96 echocardiographic exams with Doppler.	A decrease in the rate of myocardial hypertrophy and changes in cardiac function parameters were observed in the fetal and neonatal periods.
Chen et al., 2022 [8]	Evaluate cardiac geometry and contractility abnormalities in fetuses of women with well-controlled GDM using Fetal HQ.	80 fetuses of women with well-controlled GDM and 90 control fetuses.	Fetal HQ speckle-tracking technique measuring cardiac shape, global, transverse, and longitudinal contractility.	Despite good glycemic control, abnormal ventricular contractility was present in fetuses of women with GDM, but more frequent in the RV. For both the RV and LV, transverse ventricular contractility abnormality were more prevalent than abnormal global and longitudinal contractility. Fetuses of women with GDM should be evaluated for ventricular contractility abnormality and have more follow-ups despite good glycemic control.
Garcia-Flores J. et al., 2011 [15]	To make a global evaluation of the fetal myocardial changes in a well-controlled gestational diabetic population.	24 well-controlled diabetic pregnant women with well-controlled GDM vs. 16 healthy control pregnancies.	IVS thickness, cardiothoracic index, valvular diameters, myocardial function parameters.	Tendency of interventricular septum hypertrophy was observed even in well-controlled diabetic pregnancies. Mild hypertrophic cardiac changes were not associated with abnormal cardiac function or signs of left ventricular outflow obstruction, although minor changes in right ventricular diastolic function were recorded.
Halse et al., 2013 [16]	Explore relationship between serological and morphological markers of cardiac dysfunction and abnormal fetal ECG changes during labor and delivery in diabetic pregnancies.	99 pregnant women with diabetes (30 type 1, 9 type 2, and 60 GDM) and their newborn offspring.	Umbilical cord blood pro-BNP, neonatal interventricular septal thickness, fetal ECG via STAN technology.	Increased umbilical cord blood pro-BNP is associated with echocardiographic signs of cardiomyopathy and with lower umbilical cord blood pH.
Hou et al., 2021 [17]	Assess the ventricular diastolic function of fetuses exposed to GDM by looking into the diagnostic parameters using both the conventional method and Dual-gate Doppler (DD) method and to investigate the potential of DD in the early detection of fetal cardiac diastolic dysfunction.	56 women diagnosed with GDM and 55 healthy pregnant women between 24 and 30 weeks of gestation.	E/A, e’/a’, and E/e’ ratios measured via Doppler methods.	High blood glucose of women with GDM will cause impaired diastolic function in the fetuses. To assess fetal diastolic function, RV is arguably key when detecting early impairment, since alterations and damage are more likely to happen in RV. Measurement of E/e’ ratio using the DD is considered a feasible and robust method to detect fetal diastolic function in fetal cardiac diastolic function assessment. Good or poor control of the GDM does not have a significant influence on the fetal diastolic function. The early detection of GDM and GDM-induced fetal cardiac dysfunction is necessary.
Kulkarni A. et al., 2017 [11]	Assess fetal myocardial deformation in maternal diabetes mellitus and obesity using 2D speckle-tracking echocardiography (2D-STE).	178 fetuses: 82 fetuses of mothers with diabetes mellitus (FDM), 26 fetuses of mothers with obesity (FO), and 70 normal fetal controls (FC).	GLS, GCS, ALSR, ACSR using 2D-STE.	Fetal myocardial deformation was significantly altered.
Miranda et al., 2017 [9]	The aim of this study was to assess the biventricular systolic and diastolic function of fetuses exposed to maternal diabetes (MD) compared with control subjects using a comprehensive cardiac functional assessment and exploring the role of speckle-tracking to assess myocardial deformation.	129 fetuses (76 exposed to maternal diabetes, 53 controls) with structurally normal hearts examined between 30 and 33 weeks of gestation.	Cardiac morphometry, myocardial performance index, deformation parameters.	Fetuses of mothers with diabetes present signs of biventricular diastolic dysfunction and right ventricular systolic dysfunction by deformation analysis in the third trimester of pregnancy. Two-dimensional speckle-tracking could offer an additional benefit over conventional echocardiography to detect unfavorable subclinical changes in myocardial function in this population
Tejaswia et al., 2020 [13]	Investigate fetal cardiac changes in diabetic mothers and controls from 24 weeks to the neonatal period correlating with maternal glycemic control and adverse perinatal/neonatal outcomes.	185 pregnant women (83 diabetics: 17 overt DM, 66 GDM; 102 healthy controls), studied from 24 weeks to neonatal period with serial fetal echocardiography.	Echocardiographic assessment of IVS thickness, RV/LV structure, E/A ratio, Tei index, annular velocities.	Significant association between fetal myocardial hypertrophy and maternal glycemic control among GDM pregnancies. There is an association between fetal myocardial hypertrophy and some adverse perinatal events, including hypoglycemia. However, these newborns were not found to have clinically relevant cardiac comorbidities even though there was significant septal hypertrophy in utero.
Yu et al., 2019 [7]	Evaluate the associations between maternal diabetes diagnosed before or during pregnancy and early-onset cardiovascular disease (CVD) in offspring over four decades.	2,432,000 liveborn children in Denmark (1977–2016), excluding congenital heart disease cases.	Early onset CVD diagnosis, hospital admission data, maternal diabetes history, Cox regression analysis.	Offspring of diabetic mothers had a 29% increased risk of early-onset CVD, especially those with maternal CVD or diabetic complications.

Bogo M.A. et al. recently conducted a study examining the cardiac effects of GDM in fetuses and newborns, and demonstrated that despite good maternal glycemic control, 29% of fetuses exhibited myocardial hypertrophy. Even though this rate significantly decreased to 6% postnatally, shortening fraction was impaired in 6% of newborns, but none displayed clinical signs of congestive heart failure, indicating the dysfunction remained subclinical. Both the right ventricular and left ventricular myocardial performance index (MPI) increased substantially from fetuses to newborns, signifying worsening myocardial function after birth. Additionally, the study observed worsening diastolic dysfunction postnatally, as indicated by alterations in the mitral and tricuspid E/A ratio. These findings suggest that fetal cardiac changes evolve after birth, necessitating routine echocardiographic screening for newborns of diabetic mothers [10].

In another study by Garcias-FLoers J. et al., using a case–control design with 24 diabetic pregnancies and 16 normal controls, it was found that IVS thickness was significantly increased in the diabetic group (3.93 mm vs. 3.05 mm, *p* < 0.001), indicating hypertrophic remodeling. Furthermore, the tricuspid E/A index was lower (0.72 vs. 0.78, *p* = 0.03), suggesting subtle diastolic dysfunction of the right ventricle. While gestational diabetes may induce mild hypertrophic cardiac changes, its functional impact remains unspecified. Given the variability of fetal myocardial response to maternal glycemic control, further studies should explore alternative glycemic indicators beyond HbA1c for better risk stratification and neonatal cardiovascular assessment [15].

In addition, the study of Mohsin M. et al. investigated the impact of GDM on fetal cardiac function. A cohort of 50 fetuses from GDM mothers was compared to 50 controls, revealing significant alterations in cardiac function. Diastolic dysfunction was evident, with fetuses of GDM mothers revealing a decreased mitral E/A ratio (*p* < 0.001) and an elevated MPI, indicating impaired myocardial relaxation and contractility. Notably, left atrial shortening fraction was significantly reduced in GDM fetuses, especially in those presenting with MH, suggesting increased atrial pressure and compromised ventricular compliance. The study posits left atrial shortening fraction as a potential non-invasive marker for assessing fetal diastolic dysfunction, alongside conventional indices like MPI. These insights emphasize the critical need for advanced prenatal cardiac surveillance of fetuses of GDM mothers for the early detection of structural and functional abnormalities [14].

Recently, a study was conducted by Hou Q. et al. examining the impact of GDM on fetal cardiac diastolic function. Increased placental vascular resistance was identified as a contributing factor, leading to compromised myocardial relaxation and diastolic dysfunction [17]. This study revealed that fetuses exposed to GDM had significantly reduced E/e’ ratio, particularly in the RV, underscoring that E/e’ ratio could be a sensitive biomarker affected by intrauterine metabolic stressors [16]. In addition, the study indicated that RV function appears disproportionately impaired in comparison to LV function, which could be attributed to the RV’s predominant role in fetal circulation, as it provides approximately 60% of the combined fetal cardiac output [17].

Miranda J.O. et al. examined the effect on the fetus’s heart development when exposed to an intrauterine diabetic environment. It was noted that the fetuses of GDM mothers exhibited both biventricular diastolic dysfunction and right ventricular systolic dysfunction. It was also noted that the intraventricular septum was thickened in fetuses exposed to diabetes. Through the use of speckle-tracking echocardiography, significant reductions were detected in early and late diastolic strain rates in both ventricles, suggesting impaired myocardial relaxation [9]. The study also assessed right ventricular systolic function, finding that fetuses in the GDM group exhibited significantly lower global longitudinal strain in the right ventricle. Since the right ventricle contributes approximately 60% of fetal cardiac output, any dysfunction in this chamber could have considerable physiological consequences. While conventional echocardiographic assessments showed no notable differences in conventional systolic and diastolic function indicators, speckle-tracking was able to reveal subclinical signs of cardiac dysfunction that might be overlooked with standard echocardiography [9].

Furthermore, Bhorat I.E. et al. examined myocardial function in fetuses of poorly controlled GDM pregnancies. The cohort consisted of 29 diabetic pregnant women, matched with 29 controls, revealing significantly elevated modified MPI (Mod-MPI) (0.59 vs. 0.38, *p* < 0.0001) and decreased E/A ratio (0.65 vs. 0.76, *p* < 0.0001) in the diabetic group. Importantly, a Mod-MPI ≥ 0.52 demonstrated 100% sensitivity and 92% specificity in predicting adverse perinatal outcomes, which included stillbirth, neonatal death, acidosis, and cardiomyopathy, affecting 58% of the diabetic cohort. Contrarily, conventional fetal monitoring techniques such as cardiotocography and umbilical artery Doppler velocimetry failed to detect impending fetal distress in some cases. The authors suggested that Mod-MPI and E/A ratio may serve as critical cardiac fetal surveillance tools in diabetic pregnancies, necessitating further large-scale studies to validate their clinical utility [12].

The study of Halse K.G. et al. undertook a comprehensive evaluation of cardiac dysfunction in neonates born from diabetic mothers, assessing both serological and morphological indicators alongside fetal ECG monitoring during labor. In the study, 99 pregnant women with diabetes (30 with type 1, 9 with type 2, and 60 with GDM) were included. The newborns were examined using a combination of umbilical cord blood pro-brain natriuretic peptide (pro-BNP) levels, echocardiographic measurements, and fetal ECG with STAN technology. Higher pro-BNP concentrations in umbilical cord blood were strongly associated with greater neonatal interventricular septal thickness (*p* = 0.025), which suggests a link between elevated cardiac stress markers and hypertrophic myocardial remodeling. Additionally, lower umbilical cord blood pH values correlated negatively with pro-BNP levels (*p* = 0.036), reinforcing the notion that metabolic acidosis may be tied to increased fetal cardiac strain [16]. Abnormal fetal ECG changes (STAN events) showed no significant correlations with either pro-BNP levels or neonatal interventricular septal hypertrophy, signifying that while ECG alterations might reflect fetal cardiac distress, they may not serve as sensitive predictive markers of structural myocardial remodeling in neonates [17].

Additionally, the study of Kulkarni A. et al. evaluated how GDM and obesity can affect fetal myocardial function, utilizing two-dimensional speckle-tracking echocardiography to assess cardiac deformation patterns in utero. It was suggested that fetuses of diabetic mothers exhibited significantly lower global longitudinal strain and circumferential strain and strain rate compared to healthy fetal controls, indicating early signs of myocardial dysfunction [11]. Importantly, maternal obesity alone could adversely influence fetal myocardial function, with fetuses of obese mothers displaying abnormal global longitudinal strain and circumferential strain rate independent of diabetes. Clinically, the study emphasizes the importance of advanced echocardiographic techniques like 2D-STE for detecting early myocardial dysfunction in high-risk pregnancies [11].

### 3.5. Long-Term Cardiovascular Risks for Offspring of Diabetic Mothers

**Figure 2:**
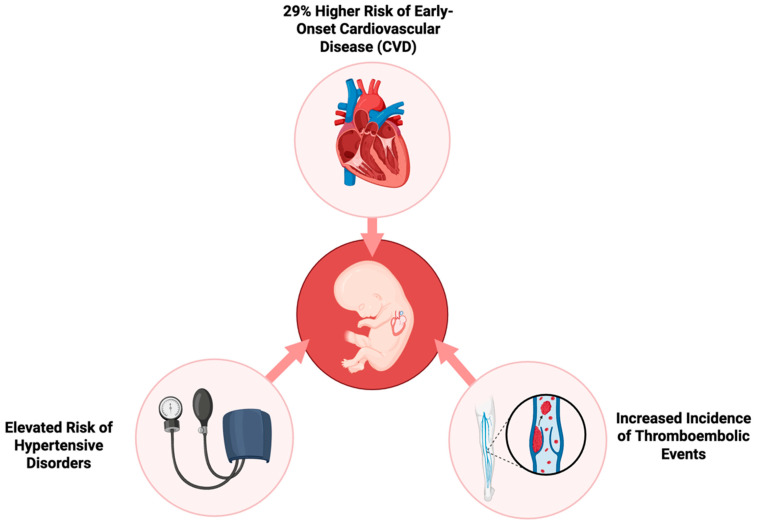
Long-term cardiovascular risks in offspring of mothers with gestational diabetes.

On the other end, Yu Y. et al. provide compelling evidence regarding the long-term effects of GDM on the cardiovascular health of progeny. The researchers employed a large population-based cohort to monitor the development of cardiovascular disease (CVD) in early life. They followed over 2.4 million children for up to 40 years to investigate the impact of prenatal exposure to GDM, whether it was present in pregestational (Type 1 or Type 2) or gestational stages [7]. The results indicate that the risk of early-onset CVD in the children of mothers with diabetes is 29% greater compared to children of non-diabetic mothers. This risk was consistent across different varieties of diabetes, with pregestational diabetes exhibiting a slightly greater impact than gestational diabetes. Hypertensive disease, deep vein thrombosis, and pulmonary embolism were more common in progeny of mothers with diabetes, suggesting that prenatal exposure to maternal hyperglycemia may have long-term effects on systemic inflammation and vascular function. These elevated risks of CVD, demonstrated in Figure 2, were already detectable in childhood and persisted into early adulthood [7]. The risk was substantially increased in cases where maternal diabetic complications or a maternal history of CVD was present. The offspring of mothers with diabetic complications displayed a 60% higher risk of developing CVD at an early age, thus suggesting that severe maternal diabetes and inadequate glycemic control during pregnancy may increase the susceptibility of the fetus to future cardiovascular disorders [11].

## 4. Discussion

### 4.1. Fetal Diagnostic Imaging Biomarkers

**Figure 3:**
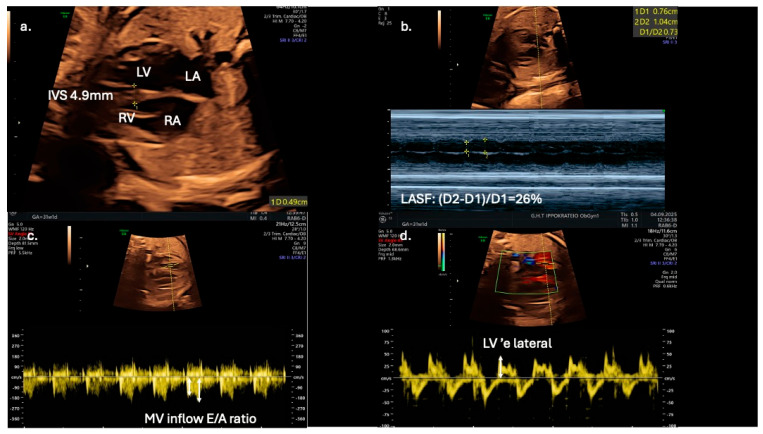
Echocardiographic indices in a 31-gestational-week fetus of a diabetic mother; (**a**) interventricular septal thickness (IVS) measured in the four-chamber view; (**b**) in the same view, left atrial systolic shortening (LASF) is calculated using M-mode echocardiography; (**c**) use of pulse wave Doppler imaging in mitral inflow to calculate E/A ratio; (**d**) application of tissue Doppler imaging in the lateral wall of the left ventricle enables further assessment of diastolic function by calculating ‘e wave and E/’e ratio.

From a clinical perspective, fetal echocardiography may serve as an adjunct tool for predicting neonatal metabolic complications. Fetal IVS hypertrophy demonstrates a strong association with increased risk of neonatal hypoglycemia, hyperbilirubinemia, prolonged admissions to intensive care units, and persistent fetal shunts (e.g., patent ductus arteriosus and patent foramen ovale). However, fetal cardiac dysfunction, as evaluated by Doppler and TDI, does not reliably predict adverse perinatal events such as intrapartum fetal distress or perinatal asphyxia [13]. Speckle-tracking echocardiography is showcased as a more sensitive tool for detecting subtle fetal cardiac dysfunction, and its assessment should be considered for all fetuses of mothers with diabetes, even in the absence of septal hypertrophy, which may appear later as a less sensitive marker (Figure 3).

Early detection of diastolic dysfunction could also have significant implications for postnatal cardiovascular health [9]. Left atrial shortening fraction could serve as an adjunct novel echocardiographic parameter for evaluating diastolic function in fetuses of diabetic mothers and may improve prenatal cardiac monitoring and management (Figure 3). In addition, Mod-MPI and E/A ratio could be utilized as early biomarkers of distress, as they are possibly more sensitive than conventional tools, such as cardiotocography and umbilical artery Doppler velocimetry. for predicting adverse perinatal outcomes in gestational diabetic pregnancies. The suggested high sensitivity and specificity thresholds (100% and 92%, respectively) for Mod-MPI ≥ 0.52 shown by Bhorat I.E. et al. highlight the need for further validation of its potential clinical utility in risk stratification and surveillance protocols [12].

Prospective investigations should focus on externally validating these promising findings in larger, multi-center prospective cohorts. Incorporating conventional diastolic dysfunction indices, and novel imaging tools such as Mod-MPI and speckle-tracking echocardiography into routine obstetric surveillance may enhance the predictive accuracy for adverse fetal outcomes and aid in optimizing perinatal decision-making for high-risk pregnancies.

Although advanced echocardiographic modalities such as speckle-tracking and dual-gate Doppler provide superior sensitivity in detecting subtle myocardial dysfunction, their clinical integration is limited. Their availability remains uneven across healthcare systems, with many centers lacking the necessary equipment or trained personnel. Even when accessible, inter-operator variability and the absence of standardized acquisition protocols reduce reproducibility and external validity. Furthermore, the cost of implementing these techniques at the population level raises questions about feasibility, particularly in resource-constrained settings. Future research should therefore not only validate the diagnostic accuracy of these modalities but also address their cost-effectiveness, scalability and training requirements. Without such considerations, the risk is that these promising tools will remain confined to specialized centers, limiting their impact on broader maternal–fetal care.

**Figure 4:**
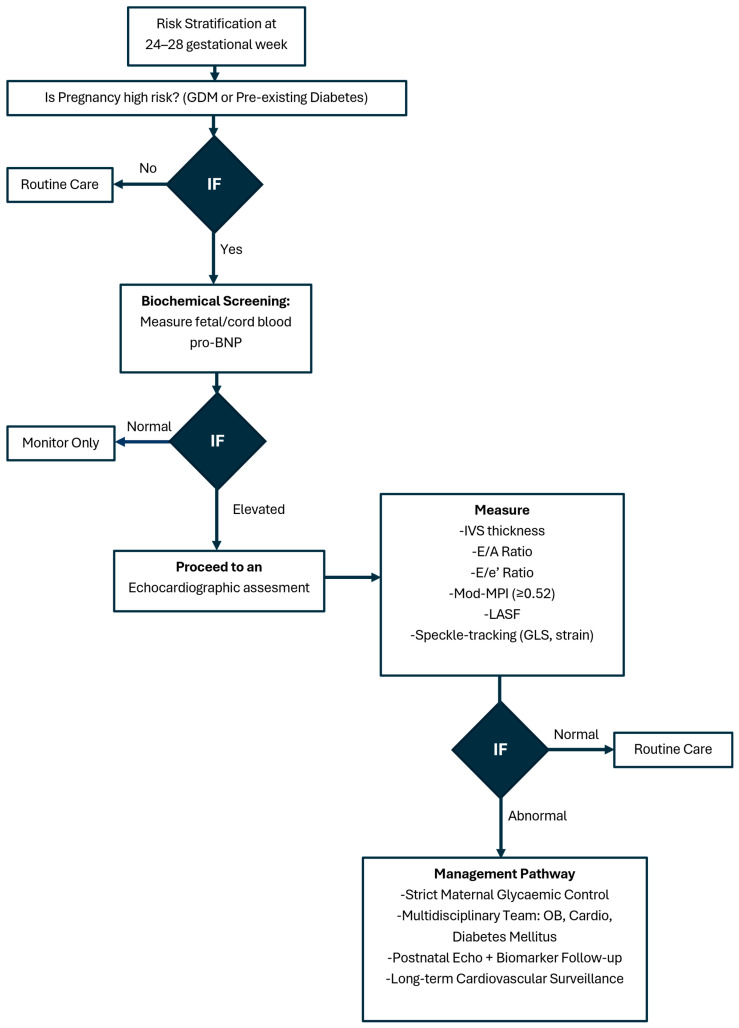
Fetal cardiac imaging algorithm in gestational diabetes mellitus.

Figure 4 presents a suggested algorithm for surveillance fetal cardiac imaging in GDM.

### 4.2. Biomarkers for Neonatal Cardiomyopathy

Regarding biochemical biomarkers, Halse K.G et al. interestingly showed a link between elevated pro-BNP measured from umbilical cord blood and hypertrophic myocardial remodeling. This suggests that pro-BNP could be used as a screening tool to identify excess cardiac stress and select patients for further assessment with imaging biomarkers. However, there is a need for further large-scale investigations to determine the role of pro-BNP in this scenario as an early biomarker for neonatal cardiomyopathy, and explore whether it could guide clinical management strategies for optimizing perinatal outcomes in high-risk pregnancies [16].

### 4.3. Optimal Maternal Glycemic Control

Optimal maternal glycemic control is essential, as even borderline hyperglycemia may be associated with significant myocardial hypertrophy and an elevated risk of neonatal morbidity. Given the ease of measurement and strong correlation with neonatal complications, fetal IVS thickness at term could serve as a simple yet effective clinical parameter for identifying neonates at risk. Despite the observed structural myocardial changes, the absence of persistent neonatal cardiac dysfunction in most cases suggests either that in utero compensatory mechanisms may mitigate long-term functional impairment, or that these subtle cardiac abnormalities are dependent on the hyperglycemic intrauterine environment and are reversible once this changes with labor [17].The significance of managing and preventing diabetes in women of reproductive age is emphasized by the study of Yu Y. et al., 2019 [7]. Optimized glycemic control, lifestyle modifications, and screening programs have the potential to significantly reduce the cardiovascular burden for both the mother and her infant throughout their lives. The results argue in favor of the long-term cardiovascular surveillance of children who have been exposed to maternal diabetes, which would allow for the early identification and management of risk factors that may lead to cardiovascular disease or complications. Proactively addressing maternal health is important in order to mitigate the prevalence of early-onset cardiovascular disease and enhance long-term outcomes for future generations [7].

### 4.4. Limitations

Despite the significant contributions made by this systematic review, certain limitations warrant careful consideration. To begin with, the majority of studies reviewed were observational in nature, and as is common with that study design, are subject to several forms of bias, variability in design, and the lack of current standards for defining gestational diabetes. There was variability in maternal glycemic control and the timing of fetal cardiac assessment through echocardiographic assessment, and the methods used may add some variability, all of which reduce the capacity to compare findings between and across the studies included in the systematic review. Moreover, since GDM is typically diagnosed after 24–28 weeks’ gestation, well beyond the completion of fetal cardiac organogenesis at around 8–10 weeks, these studies fall short in pinpointing whether the observed cardiac changes stem directly from hyperglycemia or confounding maternal factors like obesity, insulin resistance, or dyslipidemia. Many studies contributed controlled for confounding variables; however, it should be noted that many of the studies were limited by their sample size and short follow-up evaluations, which probably reduced the statistical power of the analysis and prevented the opportunity to see more subtle associations that are likely clinically relevant.

### 4.5. Future Perspectives

Most investigations neglected comprehensive glycemic metrics such as HbA1c, mean blood glucose, or postprandial fluctuations, failing to establish quantitative links between hyperglycemia’s degree or duration and cardiac dysfunction severity. The lack of a standardized approach to using advanced imaging techniques, such as speckle-tracking echocardiography and dual-gate Doppler, raises concerns about reproducibility and external validity. This inconsistency extends to varied functional indices, like E/A ratio and strain velocity, gestational timing, device settings, and heart rate corrections, fueling high heterogeneity that precludes reliable meta-analyses or diagnostic criteria derivation, while inadequate adjustments for gestational age and fetal growth may erode interpretability. Even biochemical markers such as pro-BNP, which may be used to reflect changes in fetal cardiac physiology, await full validation. Predominantly small-sized and cross-sectional, rather than longitudinal or cohort-based, these studies offer scant insight into intra-pregnancy cardiac evolution or postnatal recovery, requiring larger, tracked cohorts to validate and advance these preliminary findings. Finally, until high-quality evidence emerges to support the observations from the systematic review from larger-scale multicenter studies, it is necessary to consider the intended preliminary status of these findings.

Looking ahead, investigations should include longitudinal, multicenter studies with standardized diagnostic and imaging techniques that evaluate the trajectory of fetal and neonatal cardiac changes that happen in conjunction with maternal hyperglycemia. More advanced echocardiographic techniques, in addition to biochemical (and possibly genetic) risk markers, may also contribute to our ability to achieve early risk identification and assist in developing more tailored monitoring plans. Further, the prolonged follow-up of children/adolescents into adulthood would help delineate the effects of intrauterine exposure to gestational diabetes on cardiovascular morbidity and the developmental course across the lifespan. Studies are also needed that assess the effects of optimal glycemic control related to maternal lifestyle change or growth restriction interventions with more precise prenatal monitoring of short- and long-term cardiac outcomes. Efforts such as these will not only help to further our understanding of the mechanisms of gestational diabetes and cardiovascular disease, but also will assist in designing prevention approaches to mitigate health risks across generations.

## 5. Conclusions

GDM poses significant risks to maternal and fetal health that are present during pregnancy but may also impact the long-term cardiovascular health of the offspring. There is good evidence that maternal hyperglycemia contributes to fetal myocardial hypertrophy, diastolic dysfunction, and impaired myocardial contractility, even in cases of well-controlled diabetes. These structural abnormalities may persist postnatally, highlighting the need for early detection and neonatal follow-up. Beyond fetal and neonatal complications, children exposed to maternal diabetes face an elevated risk of early-onset cardiovascular disease. Thus, optimal maternal glycemic control remains crucial in mitigating adverse fetal cardiac outcomes. Advanced fetal echocardiographic techniques, including speckle-tracking and dual-gate Doppler, offer valuable insights into detecting subtle myocardial dysfunction, and have the potential to guide early treatment decisions and improve risk stratification if they are incorporated in specific monitoring protocols. Given the growing prevalence of GDM and its potential intergenerational effects, a proactive approach integrating prenatal cardiac surveillance, maternal metabolic regulation, and postnatal cardiovascular monitoring is essential for improving long-term health outcomes for both mothers and their children.

## Figures and Tables

**Table 1:** Population, intervention, comparator, outcome (PICO) table.

Component	Description
P	Pregnant individuals diagnosed with gestational diabetes mellitus (GDM)
I	Presence of GDM as diagnosed by established clinical criteria
C	Pregnancies without GDM
O	Fetal heart anomalies, including structural defects and functional impairments resulting in fetal cardiomyopathy

## Data Availability

No new data were created or analyzed in this study.

## Supplementary Materials

The following supporting information can be downloaded at: https://www.mdpi.com/article/10.3390/jdb13040044/s1, Table S1 GRADE Assessment of Studies on Fetal Cardiac Function in Maternal Diabetes This table evaluates the quality of evidence using the GRADE system, assessing study design, risk of bias, inconsistency, indirectness, imprecision, and potential publication bias; Table S2 New Ottawa Scale (NOS) Evaluation of Study Methodology This table presents the methodological quality assessment of studies using the New Ottawa Scale, focusing on selection bias, comparability, exposure/outcome assessment, and follow-up methods [18].
