# Supplementary material for: Cardiac Aftermath of Gestational Diabetes—From Intrauterine Impact to Lifelong Complications: A Systematic Review
Source: J Dev Biol. 2025 Dec 8;13(4):44. doi: 10.3390/jdb13040044 (PMC12733427; doi:10.3390/jdb13040044)
Supplement: Supplementary file 1 [file jdb-13-00044-s001.zip › jdb-3937925-supplementary.pdf]

Supplementary Data: Cardiac Aftermath of Gestational Diabetes - From Intrauterine Impact to Life-Long Complications: A Systematic Review

Quality Assessment of Study using GRADE and Newcastle-Ottawa Scale (NOS)

By the use of GRADE and Newcastle-Ottawa Scale (NOS) tools it is ensured that findings are critically appraised and provide standardized criteria to determine the strength of the evidence stated.

By applying both GRADE and NOS, this methodology ensured that the findings were critically evaluated to determine the strength and reliability of the evidence supporting fetal cardiac function assessments in maternal diabetes.

For the GRADE System the following criteria were considered:

Study Design**:* Randomized controlled trials (RCTs) are inherently high-quality, while observational studies begin as low-quality.Risk of Bias**:* Methodological flaws, such as lack of blinding or selective reporting, led to downgrades.***Inconsistency**:* Variability in results across different studies reduced confidence in findings.***Indirectness**:* The relevance of the study population and interventions to clinical practice was assessed.***Imprecision**:* Small sample sizes or wide confidence intervals contributed to rating downgrades.***Publication Bias**:* Whether studies selectively published favorable results was considered.***Upgrading Factors**:* Studies with large effect sizes, dose-response relationships, and minimal plausible confounding were upgraded. Each study received a final rating of ***low, moderate, or high quality**,* influencing their impact on the overall evidence base.

For NOS the following parameters were used as the main key domains:

Firstly, the***Selection Bias**:* Representativeness of the study population, sample size adequacy, and clear inclusion/exclusion criteria. Secondly ***Comparability**:* Proper adjustment for confounders and presence of well-matched control groups. Thirdly, ***Exposure and Outcome*** **Assessment**: Use of standardized measurement techniques (e.g., echocardiography, fetal Doppler), reliability of results, and blinding of assessors. Lastly, ***Follow-Up & Longitudinal Assessment***: Presence of neonatal or long-term follow-up and handling of missing data.

Studies were assigned scores ranging from 0 to 10, with higher scores indicating greater methodological reliability. Studies scoring 8–10 points were classified as high-quality, those scoring 5–7 points were moderate-quality, and those with 4 or fewer points were deemed **low-**quality.

Quality Assesment using *GRADE and NOS System Evaluation*

GRADE System Evaluation

The GRADE system **(Supl.Table_S1)** was applied for the certainty of evidence assesment that were presented in each study stated. As all studies reviewed were observational studies, they initially started as low-quality evidence unless specific upgrading factors justified an increase in rating such as large effect sizes, dose-response relationships, or minimal confounding. Studies were then evaluated based on their study design, risk of bias, inconsistency, indirectness, imprecision**,** as well as the potential publication bias.

Supl.Table_S1 GRADE Assessment of Studies on Fetal Cardiac Function in Maternal Diabetes *This table evaluates the quality of evidence using the GRADE system, assessing study design, risk of bias, inconsistency, indirectness, imprecision, and potential publication bias*

**Criteria**	**Bogo et al. 2021** [10]	**Chen et al. 2022** [8]	**Chen et al. 2022** [8]	**Hou et al. 2021** [17]	**Kulkarni et al. 2017** [11]	**Miranda et al. 2018** [9]	**Mohsin et al. 2019** [14]	**Tejaswi et al. 2020** [13]	**Yu et al. 2019** [7]	**Bhorat et al. 2014** [12]	**Garcia-Flores et al. 2011** [15]	**Halse et al. 2013** [16]
**Study Design**	Non-RCT (low quality)	Non-RCT (case report, low quality)	Prospective Observational Study (low quality)	Prospective Observational Study (low quality)	Comparative Cross-Sectional Study (low quality)	Observational Cross-Sectional Study (low quality)	Prospective Observational Study (low quality)	Prospective Observational Cohort Study (low quality)	Large Population-Based Cohort Study (high quality)	Prospective Cross-Sectional Study (low quality)	Prospective Case-Control Study (low quality)	**Prospective Cohort Study (low quality)**
**Risk of Bias**	Very serious (-2)	No serious (-1)	No serious (-1)	No serious (-1)	No serious (-1)	No serious (-1)	No serious (-1)	No serious (-1)	No serious (-1)	No serious (-1)	No serious (-1)	No serious (-1)
**Inconsistency**	No serious (-1)	No serious (-1)	No serious (-1)	No serious (-1)	No serious (-1)	No serious (-1)	No serious (-1)	No serious (-1)	No serious (-1)	No serious (-1)	No serious (-1)	No serious (-1)
**Indirectness**	No serious (-1)	No serious (-1)	No serious (-1)	No serious (-1)	No serious (-1)	No serious (-1)	No serious (-1)	No serious (-1)	No serious (-1)	No serious (-1)	No serious (-1)	No serious (-1)

**Imprecision**	Very serious (-2)	Very serious (-2)	Very serious (-2)	Very serious (-2)	Very serious (-2)	Very serious (-2)	Very serious (-2)	Very serious (-2)	No serious (-1)	Very serious (-2)	Very serious (-2)	No serious (-1)
**Publication Bias**	Undetected	Undetected	Undetected	Undetected	Undetected	Undetected	Undetected	Undetected	Undetected	Undetected	Undetected	Undetected
**Large Effect (+1 or +2)**	Present (+1)	Present (+1)	Present (+1)	Present (+1)	Present (+1)	Present (+1)	Present (+1)	Present (+1)	Present (+1)	Present (+1)	Present (+1)	Present (+1)
**Dose Response (+1 or +2)**	Not applicable	Not applicable	Not applicable	Not applicable	Not applicable	Not applicable	Not applicable	Not applicable	Not applicable	Not applicable	Not applicable	Not applicable
**No Plausible Confounding (+1 or +2)**	Present (+1)	Present (+1)	Present (+1)	Present (+1)	Present (+1)	Present (+1)	Present (+1)	Present (+1)	Present (+1)	Present (+1)	Present (+1)	Present (+1)

**Overall Quality**	**Moderate⊕⊕⊕O**	**Low ⊕⊕ OO**	**Moderate ⊕⊕⊕O**	**Moderate ⊕⊕⊕O**	**Moderate ⊕⊕⊕O**	**Moderate ⊕⊕⊕O**	**Moderate ⊕⊕⊕O**	**Moderate ⊕⊕⊕O**	**High ⊕⊕⊕⊕**	**Moderate ⊕⊕⊕O**	**Moderate ⊕⊕⊕O**	**Moderate ⊕⊕⊕O**

Supl.Table_S2 New Ottawa Scale (NOS) Evaluation of Study Methodology *This table presents the methodological quality assessment of studies using the New Ottawa Scale, focusing on selection bias, comparability, exposure/outcome assessment, and follow-up methods*

**Study**	**Selection Bias (0–3)**	**Comparability (0–2)**	**Exposure/Outcome Assessment (0–3)**	**Follow-up & Longitudinal Assessment (0–2)**	**Total Score (0–10)**	**Quality Rating**
**Bogo et al. 2021** [10]	2	1	2	1	**6/10**	Moderate
**Chaudhari et al.**[18]	1	0	1	0	**2/10**	Low
**Chen et al. 2022** [8]	3	2	3	1	**9/10**	High
**Hou et al. 2021** [17]	2	2	3	1	**8/10**	High
**Kulkarni et al. 2017** [11]	2	2	2	1	**7/10**	Moderate
**Miranda et al. 2018** [9]	3	2	3	1	**9/10**	High
**Mohsin et al. 2019** [14]	2	2	2	1	**7/10**	Moderate
**Tejaswi et al. 2020** [13]	2	2	3	1	**8/10**	High
**Yu et al. 2019** [7]	3	2	3	2	**10/10**	High
**Bhorat et al. 2014** [12]	2	2	2	1	**7/10**	Moderate

**Garcia-Flores et al. 2011** [15]	2	2	2	1	**7/10**	Moderate
**Halse et al. 2013** [16]	3	2	3	2	**10/10**	High

Scoring Criteria

- **Selection Bias (0–3): Representativeness of the population, sample size adequacy, transparency of inclusion/exclusion criteria.**
- **Comparability (0–2): Adjustments for confounders, presence of non-diabetic control groups.**
- **Exposure/Outcome Assessment (0–3): Use of validated echocardiographic techniques, blinding of outcome assessors, reliability of measurements.**
- **Follow-up & Longitudinal Assessment (0–2): Neonatal vs. long-term cardiac follow-ups, attrition handling.**
